# Graph Theory-Based Sequence Descriptors as Remote Homology Predictors

**DOI:** 10.3390/biom10010026

**Published:** 2019-12-23

**Authors:** Guillermin Agüero-Chapin, Deborah Galpert, Reinaldo Molina-Ruiz, Evys Ancede-Gallardo, Gisselle Pérez-Machado, Gustavo A. De la Riva, Agostinho Antunes

**Affiliations:** 1 CIIMAR/CIMAR, Interdisciplinary Centre of Marine and Environmental Research, University of Porto, Terminal de Cruzeiros do Porto de Leixões, Av. General Norton de Matos s/n 4450-208 Porto, Portugal; 2 Department of Biology, Faculty of Sciences, University of Porto, Rua do Campo Alegre, 4169-007 Porto, Portugal; 3 Departamento de Ciencia de la Computación. Universidad Central ¨Marta Abreu¨ de Las Villas (UCLV), Santa Clara 54830, Cuba; deborah@uclv.edu.cu; 4 Centro de Bioactivos Químicos (CBQ), Universidad Central ¨Marta Abreu¨ de Las Villas (UCLV), Santa Clara 54830, Cuba; reymolina@uclv.edu.cu; 5 Programa de Doctorado en Fisicoquímica Molecular, Facultad de Ciencias Exactas, Universidad Andrés Bello, Av. República 239, Santiago 8370146, Chile; eancedeg@gmail.com; 6 EpiDisease S.L. Spin-Off of Centro de Investigación Biomédica en Red de Enfermedades Raras (CIBERER), 46980 Valencia, Spain; giselle.perez@epidisease.com; 7 Laboratorio de Biotecnología Aplicada S. de R.L. de C.V., GRECA Inc., Carretera La Piedad-Carapán, km 3.5, La Piedad, Michoacán 59300, Mexico; griva_2010@hotmail.com; 8 Tecnológico Nacional de México, Instituto Tecnológico de la Piedad, Av. Ricardo Guzmán Romero, Santa Fe, La Piedad de Cavadas, Michoacán 59370, Mexico

**Keywords:** QSAR, topological indices, alignment-free, bioinformatics, big data

## Abstract

Alignment-free (AF) methodologies have increased in popularity in the last decades as alternative tools to alignment-based (AB) algorithms for performing comparative sequence analyses. They have been especially useful to detect remote homologs within the twilight zone of highly diverse gene/protein families and superfamilies. The most popular alignment-free methodologies, as well as their applications to classification problems, have been described in previous reviews. Despite a new set of graph theory-derived sequence/structural descriptors that have been gaining relevance in the detection of remote homology, they have been omitted as AF predictors when the topic is addressed. Here, we first go over the most popular AF approaches used for detecting homology signals within the twilight zone and then bring out the state-of-the-art tools encoding graph theory-derived sequence/structure descriptors and their success for identifying remote homologs. We also highlight the tendency of integrating AF features/measures with the AB ones, either into the same prediction model or by assembling the predictions from different algorithms using voting/weighting strategies, for improving the detection of remote signals. Lastly, we briefly discuss the efforts made to scale up AB and AF features/measures for the comparison of multiple genomes and proteomes. Alongside the achieved experiences in remote homology detection by both the most popular AF tools and other less known ones, we provide our own using the graphical–numerical methodologies, MARCH-INSIDE, TI2BioP, and ProtDCal. We also present a new Python-based tool (SeqDivA) with a friendly graphical user interface (GUI) for delimiting the twilight zone by using several similar criteria.

## 1. Introduction

Sequence comparisons between recorded genes in databases and a new query sequence are the grounds of comparative and functional genomics. Thus, sequence similarity has been a proxy to assign a biological function to new genes and proteins as well as to set homology relationships between them [1]. Sequence similarity searches traditionally have been performed by local alignment methods based on three kinds of algorithms: (1) Dynamic programming (Smith–Waterman) [2], (2) heuristic Basic Local Alignment Search Tool (BLAST) [3], and (3) probabilistic hidden Markov models (HMM) [4]. All these algorithms score the pairwise similarity measures by using a scoring system implemented in substitution matrixes [5,6]. While the Smith–Waterman algorithm finds the optimal alignment at a higher computational cost, BLAST and HMM relate their similarity scores to statistical significance estimates in order to discard those scores that could be attained by chance [7]. On the other hand, when such pairwise alignments are iteratively applied they work as multiple sequence alignments (MSA) to evaluate the similarity/dissimilarity among a set of available “gene markers” from several organisms and can be used for phylogenetic analysis (evolutionary relationship between a group of taxa) [8].

Although alignment-based (AB) methods have been implemented in web servers which have become popular among academics and researchers [9,10,11,12], their homology predictions in terms of 3D structural conservation and functional assignments start failing when sequence similarity between the query and the reference is lower than certain limits. It has been reported as a “twilight zone”, defined in the range of 20–35% identity for protein alignments where homology detection is inaccurate [13]. The same is true when the comparison implies related sequences within the twilight zone from a variety of organisms, then MSA algorithms are not suitable to provide reliable phylogenetic inferences [14]. The boundaries of the twilight zone have been recently modified by the use of other similarity measures [15].

In addition to the handicap representing the low similarity shared by two homologous sequences for their AB detection, there are other genetic events affecting the performance of alignment algorithms. For example, when comparing genomes searching for homologous regions, such detection is negatively affected if the corresponding genomes have undergone genetic events like genetic recombination, shuffling, and horizontal gene transfer. Such genome rearrangements are at odds with the assumption of alignments algorithms considering conservation and contiguity between homologous regions [16].

Last but not least, the extensive use of the computational memory and time to align long sequences or fragments has limited the multi-genome comparison. In this sense, alignment-free (AF) methods with their associated similarity measures have come to solve many of the intrinsic handicaps of the alignment-based algorithms [17]. AF methodologies generally demand less computational resources than finding optimal alignments by dynamic programming when dealing with long fragment comparisons, namely genomes or extensive proteomes. AF approaches are not sensitive to genome rearrangements and are more suitable to detect evolutionarily conserved signal at low sequence similarity [18].

Despite several state-of-the-art reviews that have been published describing the most popular AF methods with their corresponding AF similarity measures and their successful applications in sequence comparison [16,17,18,19], a group of relatively new class of AF gene/protein features have been omitted. They are extensions of topological indices (TIs) initially defined in chemo-informatics to describe the molecular structure of organic compounds by applying graphical theoretical approaches. Thus, they have been especially applied to model physicochemical and biological activities of drug-like compounds by means of quantitative structure activity relationship (QSAR) studies. When, such molecular TIs are extended to characterize the sequence/structure of DNA, RNA, and proteins through several graphical representations, quantitative sequence (structure) function relationships can be considered as AF models for predicting structural, functional, and evolutionary signals within gene/protein families.

The most relevant and widespread bioinformatic application of the mentioned topological descriptors is the detection of remote homologs within the twilight zone of DNA/RNA and protein alignments [20,21,22,23,24,25,26,27]. The other emerging and interesting application is their integration either with the most popular AF features/methods or AB similarity measures to improve the detection of homology signals in sequence comparison [28,29,30]. Here, we present enough evidence, including from our own work, that confirms the usefulness of the AF features derived from graphical–numerical approaches in sequence comparative analyses, bringing out their performance for remote homology prediction within the twilight zone. Other relevant addressed issues are the increasing trend to combine either AF features encoding different structural information, or AF with AB measures under several mathematical/statistical frameworks to improve the sensitivity of the homology detection. Lastly, we briefly pointed out some efforts made for scaling up AF and AB features/measures to analyze large datasets.

**Figure 1:**
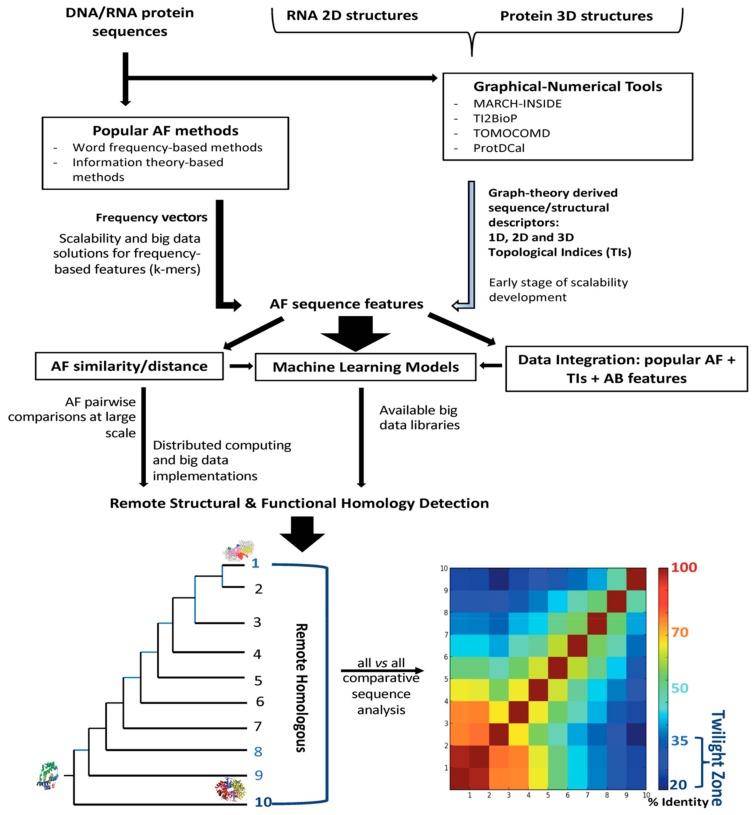
Workflow used for homology detection within the twilight zone according to the selected alignment-free (AF) methodology. This selection is conditioned in turn by the input data and the availability of scalable solutions.

Figure 1 shows an overview about the AF methodologies addressed in this review. Their applicability for the detection of structural and functional homologs within the twilight zone considering the input data is illustrated. Additionally, the availability of distributed computing and big data implementations to such methodologies is also included along the workflow for the homology detection.

## 2. The Twilight Zone for Protein and RNA Alignments

In 1999, Burkhard Rost defined the twilight zone for the protein alignments by using the pairwise sequence identity as boundary limits. Rost showed that the remote homology is hardly detected in the presence of randomly related sequences within the twilight zone of 20–35% of pairwise identity, or within the “midnight zone” placed below of 20% of identity. In the midnight zone, similar 3D structures are hard to detect with conventional alignment methodologies because they just could share 8–12% pairwise sequence identities. Many of these similar structural pairs resulted from convergent and divergent evolutionary processes misleading a correct homology prediction [13].

Despite that the homology term expresses a common evolutionary origin of structurally or functionally related proteins, it can be inferred by evaluating amino acid identities or similarities among two or more protein sequences under the supposed fact that the primary sequence of a protein determines its structure, function, and evolutionary characteristics. Therefore, Rost’s study was focused on demonstrating that similar protein structures (homologous) placed in the twilight zone were detected with inaccuracies by pairwise alignments. On the contrary, a “safe zone” was delimited, where homology was unequivocally detected by aligning at least 100 residues long sharing an identity higher than 30% [13,31].

Similar to the protein alignments, a twilight zone was more recently defined for RNA alignments considering the limit of sequence identity holding the conservation of the RNA 3D structure. Several authors have reported a twilight zone of < 50–60% sequence identity where the relationship between RNA’s sequence and structure conservation turns out weak [32,33,34].

Alignment length is a crucial factor for determining the threshold of sequence identity sufficient for detecting reliable homology [31]. Short sequences have higher chances to produce random alignments with no biological significance. This suggests that shorter sequences require more stringent cut-offs for inferring homologous relationships than longer sequences [13,35]. Despite the fact, the percentage identity of 25–35% has been traditionally considered as rule of thumb to boundary the twilight zone for protein homology identification. Currently, there is just a guidance for this aim because it is greatly affected by the length of the alignment and could underestimate the number of homologs within such zone [35]. More statistically rigorous similarity measures like the bit score and its associated e-value are being considered for assessing homology [15].

According to Pearson (2003), the BLAST bit score is a more accurate similarity measure for inferring homology than the identity percentage. The bit score measures sequence similarity independently of the query sequence length and database size. It is normalized based on the raw pairwise alignment score. Protein pairs of average lengths (not extremely long) with bit similarity scores > 50 are almost always considered significant homologs. If the search or comparison encompasses less than 7000 proteins of average length, 40 bits could be significant at e-values < 0.001 for detecting homology. Thus, we could tune up more precisely the twilight zone for protein alignments by applying the bit score similarity measure (< 40–50 bits) [1].

### SeqDivA: Sequence Diversity Analysis for Detecting the Twilight Zone

Looking into literature and bioinformatics forums, there is not a single software that can explore the diversity of a database or a sequence subset by applying reported similarity measures to delimit the twilight zone according to all previously mentioned thresholds. So far, in order to retrieve several similarity measures, like identity, similarity, and scores in an all-vs.-all pairwise sequence comparison, users should run alignment software like needle (global alignment), water (local alignment), blast (local alignment), and even multiple sequence alignments (MSAs) tools (http://imed.med.ucm.es/Tools/sias.html). Then they should parse the results to obtain a nxn matrix. However, going through all these steps to get the final similarity matrix requires specific programming skills.

**Figure 2:**
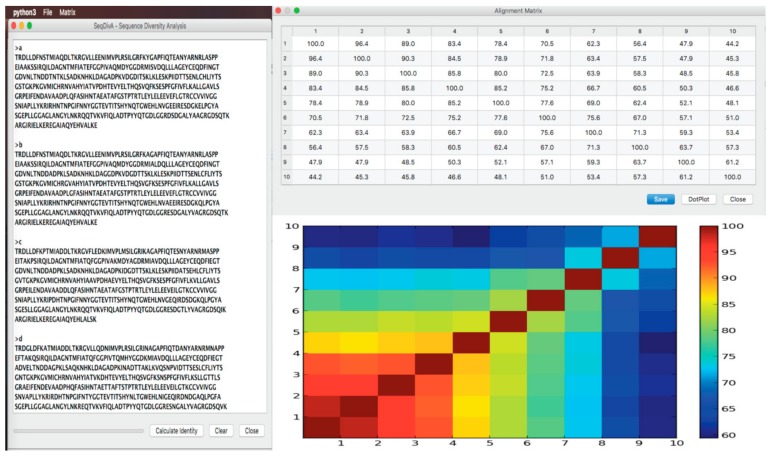
Screen shot of the SeqDivA’s GUI. The input fasta file made up by 10 hypothetical protein sequences and the main outputs: The identity matrix all-vs.-all and the dot plot representing the identity/similarly/bit-score variation among the sequence pairs.

Here, we present SeqDivA, a Python-based tool with a friendly GUI allowing non-expert users to run alignment algorithms (water, needle, and blast) to compare all-vs.-all protein, DNA, and RNA sequences (Figure 2). SeqDivA provides similarity, identity, and bit-score matrixes to explore the diversity/homology of the sequences, enabling the delimitation of the twilight zone. The resulting matrixes are visualized using dot plot-like graphs representing pairwise similarity measures (identities, similarity, and bit scores). SeqDivA also allows redundancy reduction by exploring amino acid identities from global alignments and can be connected to the output of software simulating related sequences with a known evolutionary history, i.e., ROSE - Random Model of Sequence Evolution [36] and INDELible—Insertions and Deletions simulations [37], in order to get subsets of homologous sequences at different identities or bit-scores ranges. The SeqDivA software can be freely downloaded at https://github.com/eancedeg/SeqDivA. It has been previously used to evaluate the pairwise similarity/identity of hundreds of adenylation domains and thousands of enzyme and non-enzyme protein structures (all vs. all) to identify the twilight zone [38,39].

## 3. Most Popular AF Approaches in the Twilight Zone

In this section, we compile the most popular alignment-free methods applied to the detection of homologous sequences within the twilight zone of alignment algorithms. Such homologous proteins placed at this zone or beyond are known as remote homologs.

### 3.1. Word Frequency-Based Methods

The most popular AF approaches are based on word frequency counting, known as word-based methods. They estimate how many times a letter from the DNA or protein alphabets appears along the query sequence, or alternatively they can also count the occurrences of certain subsequence of length k, where k size must be smaller than the query sequence length. Thus, they encompass those AF methods based on nucleotide [40], amino acid [41] and pseudo compositions [42], and others related to subsequence frequencies like k-mers or k-words [43], spaced k-words [44], and k-tuples [45]. They all have been applied up to a certain extent in database searching, gene annotation, comparative genomics, and phylogenetics by using AF similarity measures to cope with the previously mentioned alignment’s handicaps. For example, amino acid composition (ACC) was implemented in a webserver named composition-based protein identification (COPid) to perform protein searches and phylogenetic analysis by means of AF distances (https://webs.iiitd.edu.in/raghava/COPid/[41]) but also has been applied to detect remote homology in the G-protein coupled receptor superfamily (GPCR) [46]. The GPCR family has represented a challenging target, due to its high sequence diversity, for studying the prediction performance of several AF tools [47] including the pseudo-amino acid composition (PseAAC) protein feature [48,49].

Chou’s PseAAC concept was first applied to predict protein cellular attributes related to the biological function regardless of alignment information [50]. This AF approach incorporated the sequence order effect to the ACC to improve the quality of predictions. It was implemented in a webserver hosted at http://www.csbio.sjtu.edu.cn/bioinf/PseAA/ [51]. The performance of PseACC has been evaluated in the twilight zone by (1) identifying enzymatic signatures and delimiting their subclasses in a nonredundant subset of enzymes and nonenzymes sharing sequence similarities lower than 40% of identity [52], (2) classifying structurally characterized proteins sharing < 30% of similarity into the four Structural Classification of Proteins extended (SCOPe)’s classes (α, β, α/β, α + β) just having sequence primary information, [53] and (3) detecting remote homologous proteins using benchmark datasets [54]. In addition to the proven utility of other compositional AF features like k-mers in assembling reads from Next Generation Sequencing (NGS) technologies into contigs [55], identification of species in metagenomic samples [56,57], and improving heterologous gene expression [58], they have been applied to overcome several handicaps found in the twilight zone such as (1) the annotation of protein families within the metagenome’s diversity [59], (2) the classification of structural protein classes in designed datasets sharing low sequence similarities just by using k-word frequencies or AF distances [60,61] or by k-mers incorporation into the general scheme of PseACC [62], and (3) the phylogeny reconstruction for constantly evolving viral genomes by the estimation of alignment-free distances [63,64].

Popular AF methods based on compositional features have been also applied to genome- or proteome-based phylogeny reconstructions [65,66] because they circumvent some well-known problems arising when intending the alignment of large genomic sequences, finding orthologs to build species trees, and dealing with low homology genes/proteins [44,67]. Instead they can estimate directly AF distances from unassembled NGS reads for phylogenetic tree building [68].

Last but not least, many of the previously mentioned word-based methods have been also exploited to detect, analyze, and compare the less conserved blocks of the genomes made up by regulatory regions including promoters, transcription factors, and enhancers [69]. In this sense, the *D2z* AF measure derived from k-words frequencies highlights as one of the first reports in detecting functional and/or evolutionary similarities among cis-regulatory modules (CRMs) from several tissues of human’s and Drosophila’s genomic sequences [70]. One year later, k-words distributions were added directly to Markov models to define new AF similarity measures to discriminate functionally related CRMs from the unrelated ones [30]. In 2010, the concept of regulatory region scoring (RRS), based on the potential distribution of the transcription factors in CRMs, was introduced as an AF prediction model for the detection of related functional signals in non-alignable enhancers found in the CRMs, but could also be extended to other regulatory sequences like promoters [71]. More details about the definition and application of the most popular AF methods and measures were addressed by Vinga and Almeida in several outstanding reviews [16,17,18].

### 3.2. Information Theory-Based Methods

The runners-up of most popular AF methods are those based on the information theory which measure the information contained in the organization of DNA and protein strings using different approaches. For example, the Kolmogorov complexity of a sequence is measured through the shortest description of its string. However, such abbreviated description of the string is really expressed as a “compression” measure like the “.zip files”. As longer and more complex is the sequence, a larger description would be needed and, therefore, less compression of its string would be possible to apply [72]. Another type of complexity information measure is the Lempel-Ziv complexity that calculates the number of different substrings (occurrence rates) found along the sequence. The number of iterations needed to find such substring occurrences is related with the complexity of the sequence [73]. Once the Kolmogorov’s and Lempel-Ziv’s complexities are determined for the sequences, the estimation of similarity or distance metrics can be easily computed [72,74,75]. In this sense, compression-based distance measures from Lempel-Ziv’s and Kolmogorov’s complexities were used to detect distant protein similarities in a subset of the SCOP protein structure database [76], and to classify nonhomologous domains into the CATH levels (class, architecture, and topology) [77], respectively.

The so-called universal similarity metric introduced by Li et al. in 2001 [78] lying over the Kolmogorov complexity concept showed success to cluster protein structures sharing low sequence similarity within structural families and subfamilies [79].

Another theory-based measure is the Shannon entropy defined as the uncertainty of finding a given symbol (nucleotide or amino acid) or word (L-tuples) in the analyzed sequence [80]. The Shannon entropy concept has been used to estimate Kullback–Leibler (KL) divergence measure that allowed the comparison of two sequences [81,82]. The Shannon entropy has been recently applied to relieve the perturbation caused by several biological processes such as mutations, recombinations, insertions and deletions, and fast-evolving genomes on pairwise effective genome comparisons [83].

AF methods based on the information theory have been also applied to characterize/compare regulatory sequences [84,85] and to identify/compare transcription factor binding sites [86,87]. For further details about the application of information theory-based AF methods to noncoded sequence analysis, one may go through a comprehensive review published by Vinga [88]. At last, Table 1 shows a summary of the most popular AF methods applied to datasets of low sequence similarity for remote homology detection and the clustering of similar protein structures under such conditions.

## 4. Graphical–Numerical Approaches: Emerging AF Methodologies within the Twilight Zone

In this section, we collected a bunch of emerging methods based on graphical–numerical backgrounds addressed to the detection of homologs within the twilight zone.

### 4.1. Brief Background of Graphical–Numerical Approaches

Graph theory was first applied to characterize the complexity of small-sized organic molecules by performing a topological and combinatorial exploration of their structure. The molecular topology is represented as a graph where atoms and bonds are considered as vertices and edges of the graph, respectively. Graphs representing approximately the molecular structure can be also numerically encoded through the calculation of numerical descriptors [94,95]. Such mathematical descriptors have enriched traditional quantitative structure activity relationship (QSAR) studies for drug searches and optimizations [96].

**Figure 3:**
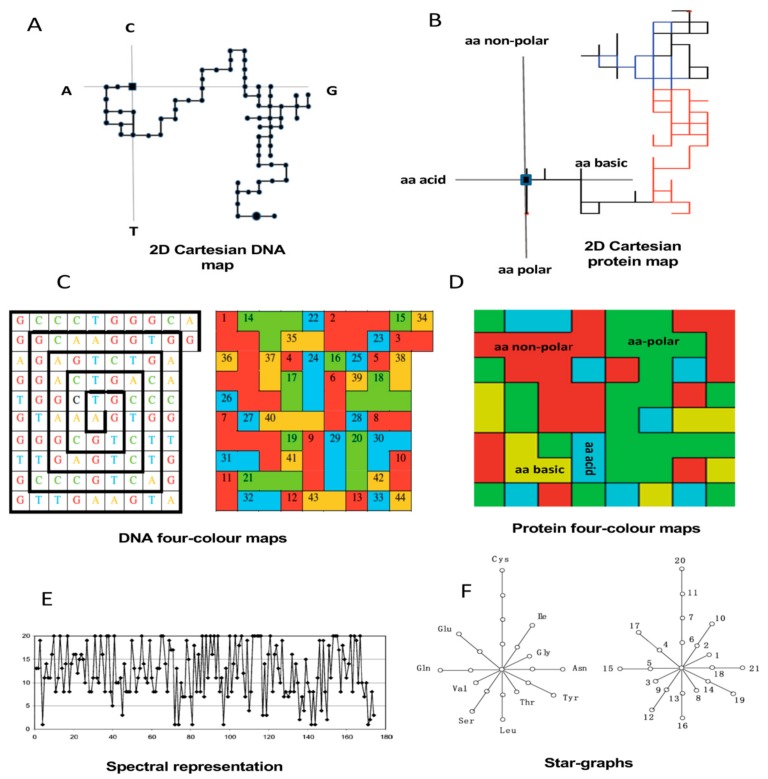
(**A**) The internal transcribed spacer (ITS2) sequence from the endophytic fungus *Petrakia* sp. pseudo-folded into the 2D-Cartesian system. (**B**) RNase III protein sequence from *Escherichia coli* BL 21 pseudo-folded into the 2D-Cartesian system extended to amino acid clustering into the four main physicochemical properties (acid, basic, polar, and nonpolar). (**C**) Representation of the human coding region of the ß-globin gene as a spiral of square cells and four-color maps [99]. (**D**) Four-color DNA maps are extended to the ß-globin protein applying the same amino acid clustering of 2D-Cartesian systems. (**E**) Spectral representation of the human ND6 protein based on the assignment of y-axis values (1–20) to the 20 amino acids. X-axis represents the length of the sequence (174 aa) [94]. (**F**) The star graph for the human insulin (21 aa long) [94,100].

**Figure 4:**
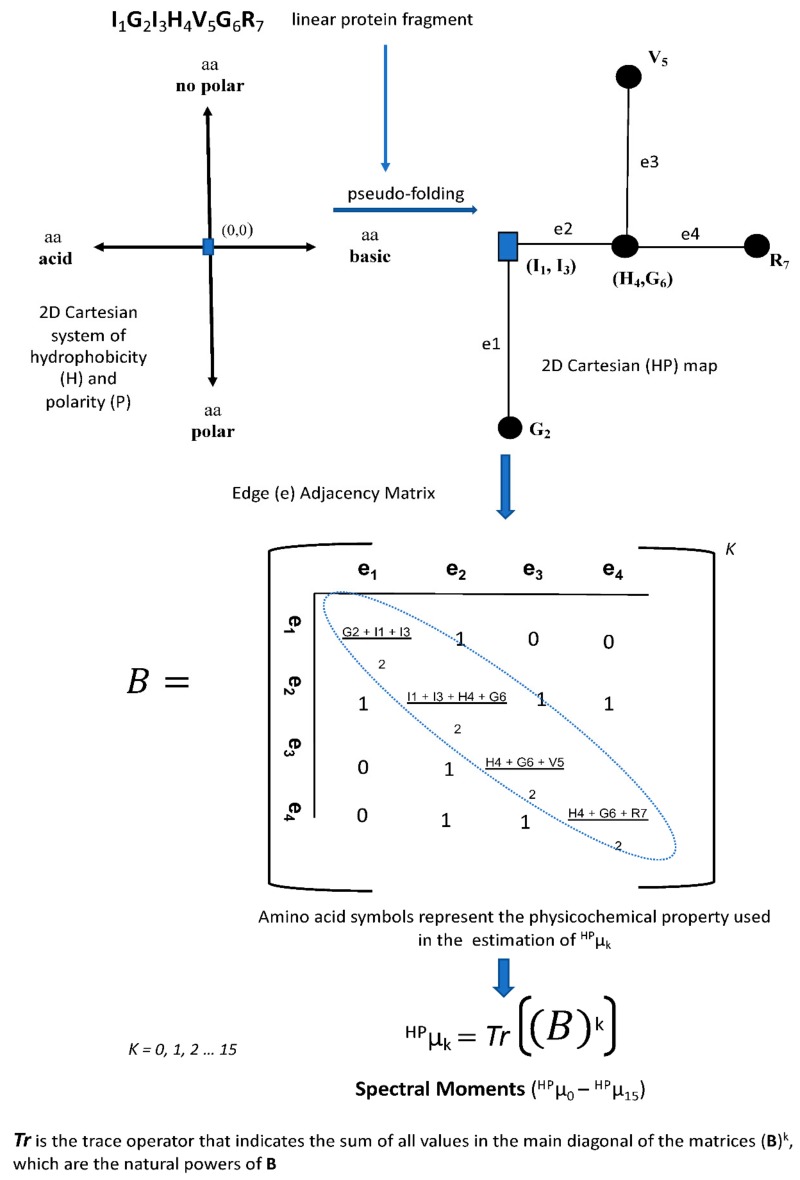
Workflow for the calculation of spectral moments as graph theory-based sequence descriptors. The protein fragment “IGIHVGR” was pseudo-folded into the 2D-Cartesian system of hydrophobicity (H) and polarity (P). The seven amino acids of the protein fragment are distributed according to their physicochemical nature into the 2D-Cartesian system starting from the 0,0 coordinates. The resulting 2D-Cartesian (HP) map is used to derive an edge adjacency matrix which is raised at different k powers. The trace operator (Tr) is applied to each (matrix)^k^ to finally estimate the spectral moments as protein TIs.

More recently, graph theory has been extended to characterize DNA and protein structures for performing comparative analysis with no alignments. Long and complex biopolymers can be simplified into 2D and 3D artificial representations of their structures. Such 2D and 3D graphs do not represent the real structure of DNA, RNA, and proteins but are expected to be useful for exploring pairwise similarities and differences by extracting hidden patterns of the underlying sequences [94,97]. The 2D artificial representations have been more widespread due to their relative simplicity for qualitative (visual) inspection (Figure 3) and for deriving numerical descriptors from topological distance matrixes representing the 2D graphs [94,97,98] (Figure 4).

Regardless of the sequence representation type, the definition of a topological matrix is mandatory for the calculation of any numerical descriptor. There are variants of the topological matrixes representing connectivity/adjacency relations between nodes/edges in the graph. For biological sequences, nodes represent the nucleotides or amino acids and edges the nucleotide or peptide bonds, respectively. In addition to the topological matrix variants, several algorithms may be applied on the topological matrixes to provide a diversity of sequence numerical descriptors. Figure 4 shows a workflow illustrating how an adjacency matrix is derived from a protein 2D graph and an operator is applied to such matrix to definitively give the protein numerical descriptors or topological indices (TIs).

For more information about the graphical and numerical characterization of DNA and protein sequences, two comprehensive reviews were published by Nandy et al. [97] and Randić et al. [94], respectively.

Graphical–numerical approaches have been applied to encode the structure of DNA, RNA, and proteins for functional annotation with no alignments [101,102,103], for the prediction of the stability of Arc repressors [104,105], the interaction between drug receptor [106,107], and detection of protein biomarkers in human diseases [108]. However, the potentialities of such approaches to overcome some bioinformatics’ handicaps like the detection of remote homologs in the twilight zone have not been fully explored and that is probably the reason why they are not considered among the AF methods reviewed in recent reviews [16,17].

### 4.2. Graphical–Numerical-Based Methods in the Twilight Zone

Although, graphical and numerical approaches have been applied for comparative analyses of DNA/proteins for many years, the studies were limited to a few cases aimed to demonstrate the discriminative power of such approaches for sequence comparison. Seminal works, mainly from Milan Randić and Ashesh Nandy, have shown the potentialities of 2D artificial graphs/maps such as spectrum-like [109], star-like [100], Cartesian-type [97,110], and four-color maps [99,111] to characterize DNA/protein sequences allowing an effective visual and numerical comparison of a few cases. As mentioned before, the 2D maps resulted in more popular representations than 3D ones despite the loss of information implying the dimensionality reduction, especially for long DNA/protein sequences [94]. However, we have demonstrated that these DNA and protein maps can generally reveal higher-order useful information contained beyond the primary structure. Hence, nucleotide/amino acid frequency distributions mapped onto the 2D space and their numerical encoding allow comparing a greater number of sequences for applications in the functional genomics and phylogenetics [38,101,112,113]. Indeed, Figure 3 illustrates some of the most frequent 2D artificial representations used for characterizing DNA, RNA, or protein sequences.

#### 4.2.1. MARCH-INSIDE Sequence Descriptors

The first works applying 2D artificial representations and the associated numerical indices to massively compare/classify protein sequences were published by authors of this review together with González-Díaz H. [101]. González-Díaz and Molina-Ruiz were the main developers of the Markov chain invariants for network selection and design (MARCH-INSIDE) methodology encoding several types of topological molecular descriptors inspired on the k-th power of the electron-transition stochastic matrix weighted with Pauling’s electronegativities [114]. This matrix summarized the molecular topology (atomic connections) of organic compounds as Markov chain states representing the transition probabilities of electrons of going from the i-th atom to the j-th atom (if they are connected) at different time intervals. The atomic elements of the main diagonal (i = j) represent the self-returning probabilities values influenced by the electronegativity of neighbor elements. The sum of their values is considered stochastic spectral moments [115], in analogy to the original spectral moments defined by Estrada as the sum of the main diagonal elements of the edge adjacency matrix representing the relationship between atomic bonds in the molecular graph topology [116].

This graph-theoretical matrix was later extended to characterize peptides and protein sequences by using 1D sequence representations and the electronic charge index for the amino acids instead of electronegativities [117]. In analogy to the electron-transition stochastic matrix, the charge-transition stochastic matrix was defined to codify topology information through the charge distribution between the adjacent amino acids in the protein. However, it was modified by a vector containing the charge of each i-th amino acid normalized by the sum of the charges of all amino acids. This vector containing absolute probabilities expands the charge distribution along the protein polypeptidic backbone, not only to the covalent-bonded amino acids [117]. The entropy involved in such charge distribution at k time/intervals along the linear protein sequences was used to predict the stability of Arc mutants [117], modelling the bitter taste of dipeptides [118] and other protein biological activities [119] as well as for modelling local drug–nucleic acid complexes [120]. However, these previous graph-theoretical matrixes defined for molecules and sequences used graphical representations intending to get close to the real structure in order to correlate them to a particular physicochemical, pharmaceutical, or biological activity.

In 2006, we introduced novel stochastic descriptors, called 2D coupling numbers, that were similarly encoded to the stochastic amino acid charge distribution along the sequence but applied to 2D-Cartesian maps. The introduced Cartesian maps distribute the sequence amino acid order according to their physicochemical properties forming four groups into the 2D-Cartesian space. Each one of the four amino acid groups match with a coordinate axes of the Cartesian system (Figure 3B). In this sense, hundreds of 2D-Cartesian maps representing plant’s polygalacturonase-like proteins and other nonredundant set of functionally unrelated proteins were numerically characterized with the so-called 2D coupling numbers to develop QSAR-type models annotating polygalacturonases members with no alignments [101]. In another report, other Markovian topological descriptors, like the stochastic spectral moments and entropies, were firstly defined for the same 2D protein Cartesian maps and evaluated on the same dataset [103]. These new protein descriptors with AF models were useful to register new polygalacturonases sequences from Psidium guajava and Coffea arabica at GenBank [101,103]. On the contrary, polygalacturonase protein members probably were not the most appropriate targets for evaluating AF protein features due to their conservation degree [121]. Consequently, we are showing, below, examples about the performance of graphical–numerical approaches in remote homology prediction in highly diverse gene/protein families reaching the twilight zone.

##### The Pac1 Detection with 2D-Cartesian Maps and Stochastic Spectral Moments

**Figure 5:**
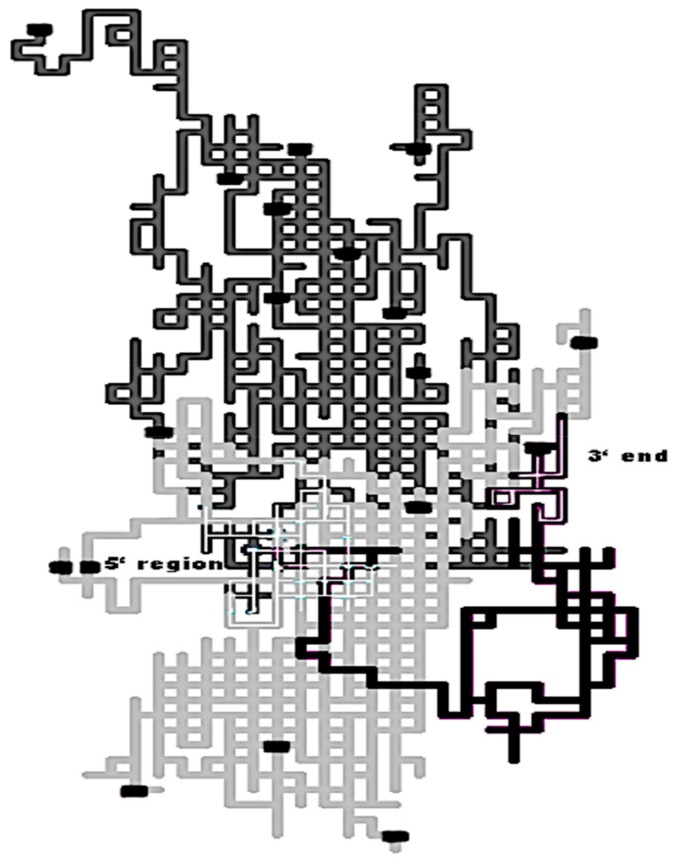
2D-Cartesian maps for several Ribonucleases (RNase) III sequences from prokaryotes (dark grey), eukaryotes (light grey), and rPac1 [DQ647826] from *Schizosaccharomyces pombe* strain 428-4-1 (black). Thin white lines represent the beginning of all RNases III (5′ region) and the terminal 3′ region of the Pac1 protein. The last amino acid from each sequence is represented as a black squared dot. This figure was taken from [122].

The first report addressing the homology detection within the twilight zone using 2D-Cartesian maps and stochastic spectral moments as TIs arrived in 2008 with the identification of the Pac1 as particular ribonuclease III (RNase III) member [122]. Pac1 is an RNase III from the *Schizosaccharomyces pombe* yeast sharing an overall low sequence similarity with typical prokaryotic and eukaryotic RNases III. The RNase III family members by themselves show a variable sequence similarity (20–84%), accounting for the low conservation of their primary structure (sequence and domain organization). Many members of this family are placed in the twilight zone such as the case of Pac1 sharing 20–40% of amino acid identities with other typical RNases III [123,124]. Nevertheless, the location of the 2D-Cartesian representation of the Pac1 protein sequence over the lattice made up of 2D-Cartesian maps representing typical prokaryotic and eukaryotic RNases III sequences revealed more structural similarities than the ones obtained with multiple sequence alignments (Figure 5). Since the Cartesian 2D protein representation is mainly based on amino acid composition, we can highlight a major region from Pac1 (in black) matching eukaryote sequences (in light gray) and another small region that lays within the prokaryote region (in dark gray). The Pac1 protein acts as a sort of bridge, linking prokaryotic and eukaryotic RNase III classes.

An AF prediction model built with these stochastic topological indices (TIs) successfully classified RNases III members (97.35%) among the sequence diversity represented by the RNase III class and the structurally nonredundant subset of the Protein Data Bank (PDB). The prediction accuracy of the AF model showed a similar performance to sensitive alignment-based algorithms like HMMs in both detecting the RNase III members of the test set and the new Pac1 [122].

##### 2D-Cartesian Maps and Markovian Entropies to Detect Remote Homologs in Cellulase Complexes

Cellulase complexes are composed mainly of three enzymes (endoglucanase, exoglucanase, and β-glucosidase) acting in a synergistic way. These enzymes are found in many fungi and bacteria species which are of great interest for bioethanol production [125]. Many years ago, the diversity of cellulase was demonstrated by performing hydrophobic cluster analysis showing the presence of subfamilies [126]. More recently we have explored the diversity of the cellulase family by performing an all-vs.-all Smith–Waterman local alignment (23–99%), and by clustering Markovian entropies encoding their sequences with 2D-HP maps (Figure 4). Both analyses showed structural variability among the cellulases and even within the three enzymatic subfamilies [127]. Three AF models were developed for each cellulase enzymatic component (endoglucanase, exoglucanase, and β-glucosidase), respectively, by using entropy measures of pseudo-folded sequences in the 2D-HP space. The classification power of the AF model for the three cellulase components was comparable to more sensitive AB methods like the HMM-profiles of Protein family (Pfam), showing an excellent performance (> 85%) at a wide range of sequence similarity.

The other advantage of our approach is that the prediction output (score) of the three models can be combined to score the whole cellulolytic complex as an alternative to the AB classification for protein enzymatic complexes or multidomain proteins [127].

##### 2D-Cartesian Maps, Star Graphs, and Markovian TIs Characterizing Mycobacterial Promoters

Because promoter sequences are among the less conserved genomic regions, Markovian indices were also evaluated to classify low-homology mycobacterial promoters using stochastics TIs derived from 2D-Cartesian and star graphs. A set of stochastics TIs (electrostatic potentials, spectral moments, and Markov entropies) were derived from 2D-Cartesian DNA representations to develop QSAR-type AF models to predict mycobacterial promoter sequences. The other Markovian TIs class was derived from star graphs (SGs). SGs are also abstract 2D representations firstly defined for proteins by Randić [108] and later extended to represent DNA/RNA sequences and proteome spectra in the S2SNet Python-based tool as a source of several types of TIs [128]. SG is an artificial 2D representation of protein sequences having an imaginary center emitting “rays” like a star. The number of rays (radial lines) are equal to the amino acid types (20 natural aa) and the nodes presented in each ray represent the frequency of each aa in the sequence (Figure 3F). For DNA, SGs are built either considering the nucleotide composition, like in proteins, or DNA codons discarding final incomplete codons. From the SG, several types of matrices (connectivity, distance, and degree) can be derived by including the original topology of the sequence or not to calculate several types of TIs. When Markov’s normalization is applied to *k*th-powered matrices, stochastic TIs are estimated among others. The AF models (QSAR-type) from 2D DNA Cartesian representations could identify promoter sequences with an accuracy > 90% considering the diversity of the overall DNA data (mycobacterial promoters and control group) while the SG’s TIs hardly got classification rates of 70% [129].

Finally, MARCH-INSIDE also encodes 3D-Markovian descriptors based on PDB coordinates considering only their alpha-carbons (Cα) for functionally annotating/classifying protein sequences in datasets marked by the low similarity. Such descriptors were evaluated in the Dobson and Doig (D&D) dataset achieving a simpler linear model able to classify the 74.18% of the proteins [130].

#### 4.2.2. S2SNet’s TIs: Star-Like Graphs Detecting Remote Enzymatic Signatures

In 2008, Munteanu C.R. et al. developed the Star Networks (S2SNet) GUI Python-based application in order to turn any sequence into 2D-SGs and deriving a variety SG’s TIs. Many of them, such as the trace of connectivity matrices, Harary number, Wiener index, Moreau–Broto indices, Balaban distance connectivity index, Kier–Hall connectivity indices, and Randić connectivity index, among others were inspired by those previously defined for describing the molecular structure of small-sized compounds. SG’s TIs were successfully applied to discriminate natural proteins from those randomly generated in silico simulating the composition and average length of the natural ones. The natural protein set was structurally well characterized sharing < 20% of homology [131].

They were also evaluated within the twilight zone by discriminating enzyme-like and nonenzyme-like protein sequences [25]. For this purpose, the previously mentioned dataset designed by Dobson and Doig (D&D) consisting in a structurally nonredundant subset of PDB members sharing < 30% of amino acid identities was used [132]. D&D dataset has been extensively used in the literature for evaluating the prediction power of AF features in the detection of remote homologs [39,52,133]. The authors concluded that SG’ protein descriptors showed similar discriminative power in classifying enzymes/nonenzymes than those encoding the 3D structure [25].

#### 4.2.3. Topological Indices to Biopolymers (TI2BioP)

The topological indices to biopolymers (TI2BioP) methodology was intended to become a practical alignment-free tool for tackling homology detection at the twilight zone and beyond it. Thus, TI2BioP should be able to deal with large datasets in order to extend its usefulness to explore genomic and proteomic data. That is the reason why TI2BioP only uses 1D and 2D representations of DNA, RNA, proteins and simple TIs to relieve the computational burden implied in the arrangement of DNA/RNA and protein strings into a 2D artificial space (called pseudo-folding) and the topological matrixes building for the final calculation of the biopolymer descriptors [22]. We recently rewrote the TI2BioP’s code in Python 3.0 for improving the graphical interface by using the PyQt5 packages and the calculation speed of TIs by optimizing the use of threads and multiprocessing implemented libraries. Such improvements guarantee more compatibility across Windows, Linux, and Mac operating systems. The latest version adds more physicochemical properties for weighting DNA/RNA nucleotides for the calculation of the TIs. TI2BioP 3.0 can be free downloaded from https://sourceforge.net/projects/ti2biop/.

In short, TI2BioP allows the pseudo-folding of DNA/RNA and protein strings into 2D-Cartesian and four-color maps and the calculation of the spectral moments defined by Estrada E. for small molecules as simple TIs [116], but here are extended to biopolymers. The 2D-Cartesian and four-color maps were introduced for DNA strings by Nandy A. [110] and Randić M. [99], respectively (Figure 3A,C), and later adapted to protein sequences by Agüero-Chapin G. et al. [38,101] (Figure 3B,D) by replacing the four nucleotides with four amino acid clusters.

The workflow previously-shown in the Figure 4 illustrates how spectral moments are estimated as TIs for the protein fragment “IGIHVGR” pseudo-folded into the 2D-Cartesian system of hydrophobicity and polarity by the TI2BioP methodology [134].

So, far we have validated TI2BioP’s numerical indices in detecting remote functional signals in the following gene/protein families: (1) Bacteriocins [134], (2) RNases III [135], and (3) internal transcribed spacer (ITS2) [20] and adenylation (A-) domains from nonribosomal peptide synthases (NRPS) [38]. These TIs have also evaluated with other AF and AB methodologies in the detection of enzymatic signatures [39] and ortholog pairs [29] within the twilight zone.

##### Bacteriocin Remote Homologs Characterized with 2D-HP Maps and Simple TIs

Bacteriocins are proteinaceous compounds of bacterial origin that are lethal to bacteria other than the producing strain. The bacteriocin protein family was targeted for testing our methodology due to its great diversity in terms of size, methods of production and killing, genetics, microbial target, immunity, etc. Such diversity is also presented in the primary structure by showing a low pairwise sequence similarity (23–50%) [136]. Thus, bacteriocin identification is a challenge for alignment algorithms which have been forced to apply complex strategies to tackle the twilight zone [137].

Since hydrophobicity and basicity are the major criteria for the antibacterial activity detection of bacteriocins, we pseudo-folded bacteriocins sequences into a 2D-Cartesian map called the 2D-HP space distributing the 20 natural amino acids into four groups according to their hydrophobicity (H) and polarity (P) properties (polar, nonpolar, acidic, or basic amino acid). For more information about the 2D-HP space for protein sequence arrangements, see the references [122,134] and Figure 4.

**Figure 6:**
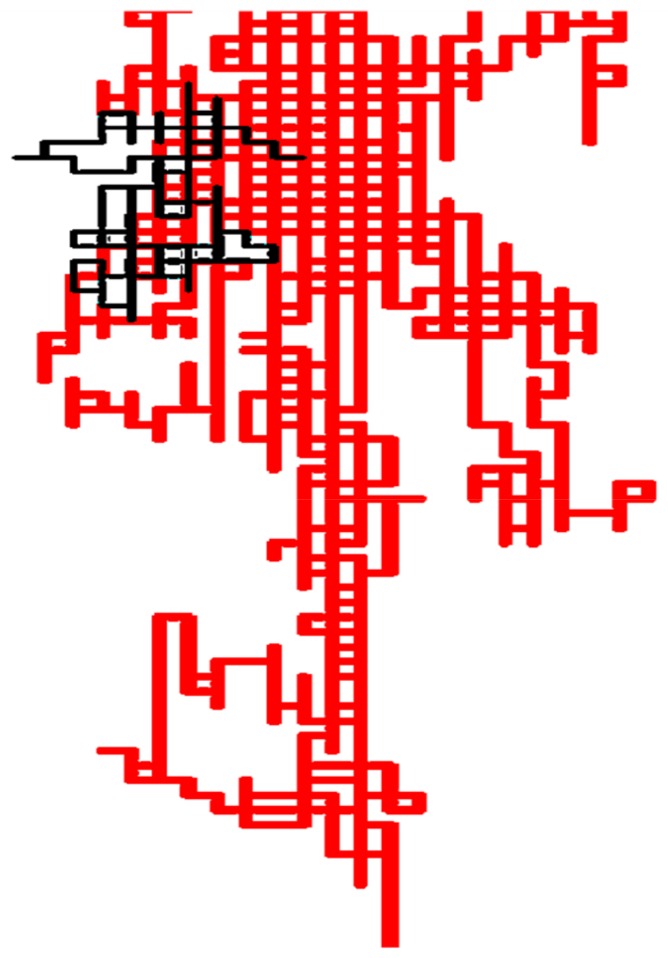
Pseudo-folding of the Cry 1Ab C-terminal domain sequence (in black) into the bacteriocins 2D-HP space (in red). This figure was taken from the reference [134].

Once protein bacteriocin sequences were arranged into the 2D-HP space, spectral moments series were calculated for the first time to build a simple linear AF model that identified the 66.7% of the bacteriocin-like proteins from an external test set, while the InterProScan could just detect 60.2% [138]. Most of the hits were detected within the twilight zone. This model was able to detect a very remote homology relationship between bacteriocins and the Cry 1Ab C-terminal domain from *Bacillus thuringiensis’s* endotoxin not previously detected by alignment methods. Although bacteriocins and Cry 1Ab C-terminal sequences are completely different, and therefore placed in different protein classes according to similarity-based searches, both share common biological features and function [134]. The bactericide function of the Cry 1Ab C-terminal domain from *Bacillus thuringiensis’s* endotoxin was only unraveled by experimental procedures in a previous report, also co-authored by some researchers of this review [139]. Homology relationship was in silico revealed only by the superposition of the 2D-Cartesian maps for Cry 1Ab C-terminal domain to other representative bacteriocins (Figure 6) and by the AF model built with our graph theory-based protein descriptors.

##### RNase III Diversity Characterized by 1D and 2D Amino Acid Clustering Strategies

Simple spectral moments derived from the 20 amino acid clustering according to their physicochemical properties into a 2D-HP space (2D-Cartesian maps) were also applied to detect RNase III members, a protein family previously targeted for evaluating Markovian protein indices by using the same representation. As mentioned before, the RNases III share pairwise similarities ranging from 20% to 84%. Such structural diversity has led to a subdivision of the enzymatic class into four subclasses represented by four archetypes (bacterial RNase III, fungal RNase III, Dicer, and Drosha) placing the most distant members at the twilight zone. For the first time, we report a simple and interpretable decision tree model (DTM) to identify RNase III members throughout their diversity and that of the nonredundant subset of the PDB made up of enzymes and no enzymes. The DTM showed a high predictive power (96.07%) using spectral moments as input predictors [135]. As the strategy of the 20 amino acid clustering into four HP classes in a 2D-Cartesian space have worked for remote homology detection either by comparing graphical profiles of representative members of the family or by deriving AF predictive models to detect family signatures, we also extended this approach to generate more predictive HMM profiles. A nonclassical HMM for this family was constructed by grouping the amino acids according to their charge values and thus reducing the alphabet of the protein sequences used as the training set to five characters corresponding to the group (group I = A, S, G; group II = M, L, I, V; group III = K, R, T, H; group IV = N, D, E, Q; and group V = F, Y, W), respectively. Regardless of their charge characteristics, proline and cysteine remained unchangeable, due to their biological meaning. This nonclassical HMM profile showed the highest prediction rate (100%) for the RNase III class regarding all previously reported AF models, either the ones built with 2D stochastic or the other nonstochastics TIs. However, the easy usability of the DTM in respect to the nonclassical HMM was illustrated by predicting a new bacterial RNase III class member isolated, enzymatically tested, and registered by our group [135].

##### Internal Transcribed Spacer (ITS2) Region

TI2BioP was also applied to identify the ITS2 genomic region in eukaryotic species. The ITS2 region was placed between the conserved 5.8S and 28S rDNA genes showing a high degree of variation even between closely related species. This particularity has allowed its application for fungal species identification at low taxonomic ranks (genus and species level) and for elucidating phylogenetic relationships among closely related genera and species. Conversely, the high sequence divergence of ITS2 has complicated its annotation by alignment algorithms and limited its use to the taxonomic identification and phylogenetic analyses at low taxonomic ranks [140]. ITS2 sequence diversity was confirmed by all-vs.-all pairwise identities/similarities provided by global and local alignments. Most of the pairs were placed at the twilight zone for DNA/RNA alignments (< 50–60%) [20,34].

Since the ITS2 secondary structure was more conserved among all eukaryotes than sequences, we applied two types of 2D representations for DNA sequences, the 2D Cartesian map and other derived from folding thermodynamics rules from the Mfold software [141]. These 2D maps characterized 4355 ITS2 sequences and a negative set made up of 14,657 untranslated regions (UTRs) of eukaryotic mRNAs. TI2BioP’s TIs were derived from each type of 2D maps and served to develop artificial neural networks (ANN)-based models for the ITS2 classification. The performance of both ANN-models (2D-Cartesian and Mfold) achieved classification accuracies higher than 95%, outperforming sensitive alignment-based methods like the HMMs generated with different MSA algorithms including those optimized for the set of low overall sequence similarity [20].

In order to illustrate the relevance of our approach, a new ITS2 sequence from an endophytic fungus isolated by our group was assessed in the previous AF models and used in phylogenetic analyses using AF similarity measures to aid the taxonomic classification of the targeted fungus. Our fungal isolate was placed into the *Petrakia* genus according to its morphological features, but traditionally members of this genus are hard to taxonomically place. In fact, the Taxonomy database of the National Center for Biotechnology Information (NCBI) does not provide an exact classification for the higher taxonomic ranks (class and subphylum) at what *Petrakia* genus belongs to. Assuming that our fungal isolate belongs to the Pezizomycotina subphylum according to “The Dictionary of the Fungi” [142], then a higher-level phylogenetic analysis was carried out to elucidate the class of *Petrakia* sp. by using AB and AF distance trees. Both trees, the traditional AB and the AF clustering, placed our *Petrakia* isolate within the Dothideomycetes class, confirming that our graphical–numerical approach extracts relevant biological information with an evolutionary significance [20,129].

##### Four Color-Maps and Simple TIs Characterizing NRPS’s A-Domains Diversity

A-domains are mandatory in each NRPS’s module because they are responsible for the selection and activation of the amino acid to be incorporated in the growing peptide chain during the nonribosomal peptide synthesis. However, A-domains show a great variability, mostly ranging from 10–40% of sequence identity among the NRPSs. Consequently, homology detection among the NRPSs is not a simple task. In fact, A-domain members cannot be easily retrieved by BLASTp (blast searches against protein database) by using a single template [143].

In this sense, DNA four-color maps defined by Randić [99] were adapted to characterize A-domain protein sequences by grouping the amino acids according to their physicochemical properties into four classes, as in 2D-HP maps [38]. Then, each amino acid group was assigned to a color in the four-color map. In addition to the graphical depiction of the A-domain sequences as four-color maps, spectral moments were derived from them for the first time as TIs to build AF models based on machine learning (ML) techniques for functional annotation. Among the ML-based models built with the spectral moments, a decision tree model (DTM) was selected due to its high classification rate and simplicity to detect A-domains in a highly diverse dataset. The DTM built up with four-color maps TIs outperformed popular AF approaches like ACC and PseACC, showing the highest sensitivity and the minimum of false positives in A-domains identification. It also competed with the performance of sensitive AB algorithms like the HMM profile and the multi-template BLASTp [38].

**Figure 7:**
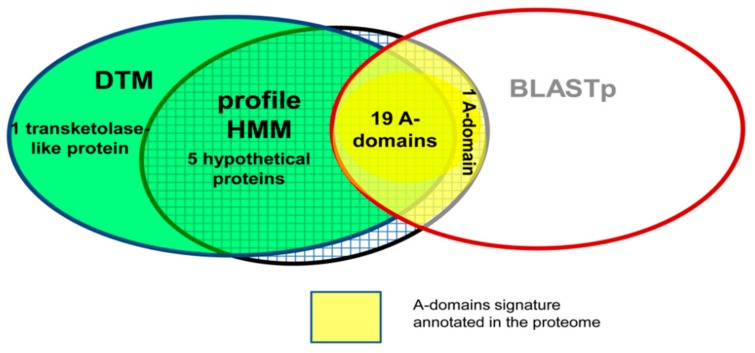
Re-annotation of the A-domains in the proteome of *Microcystis aeruginosa* by using an ensemble of algorithms. Five putative A-domain remote homologs were consensually detected by the Decision Tree Model (DTM) and the profile Hidden Markov Model (HMM) among the five hypothetical proteins. This figure was taken from the reference [38].

Additionally, the DTM was applied in cooperation to AB algorithms (multi-template BLASTp and HMMs) to fully re-explore A-domain signatures in the proteome of the cyanobacteria *Microcystis aeruginosa* NIES-843 (Figure 7). Putative A-domain remote homologs were commonly detected by the profile-based methods (DTM and HMMs) among the hypothetical proteins (green squared area of Figure 7) while the 20 annotated A-domains in the proteome of *Microcystis aeruginosa* were commonly detected by two methods (HMM and multi-template BLAST). Look at the common yellow area of Figure 7 to see that just DTM misclassified one hit. Nonetheless, the multi-template BLASTp could not detect additional A-domain signals.

In this cooperative search, DTM is an AF model built with TIs derived from 2D graphical profiles of the A-domain sequences and the HMM relies on MSA profiles. Consequently, the assembling of profile-based methods extracting different protein structural features give a better description of the A-domain signature capturing remote signals within the diversity of the proteome [38].

#### 4.2.4. TOpological MOlecular COMputer Design (TOMOCOMD) Descriptors

Marrero-Ponce et al. extended their 2D and 3D molecular descriptors defined for organic compounds in the TOMOCOMD-CARDD (computed-aided ‘rational’ drug design) software to bioinformatics and protein science (http://tomocomd.com/software).

The 2D global and local molecular descriptors implemented in the TOMOCOMD-CARDD were originally defined to characterize the structure of organic compounds for the prediction of physicochemical properties and for rational drug design by using mainly QSAR techniques [144,145]. In general, TOMOCOMD’s 2D descriptors were also derived from the molecular topology described by adjacent Cα connections and noncovalent interactions depicted as diverse graphical schemes. Then, linear, bilinear, and quadratic algebraic forms were applied to numerically describe such molecular graphs in different ways. Although these 2D molecular descriptors have been extended to bioinformatics by modelling the interaction between RNA and drugs [146] and by predicting the stability of a set of Arc mutants [147] even using novel bilinear algebraic forms [148], they have not been challenged to detect remote homology in the twilight zone of protein alignment yet. In this sense, only more recent 3D biomacromolecular descriptors defined by Marrero-Ponce et al. were assessed to classify protein sequences into the four major structural classes (α, β, α/β, α + β) by using the dataset proposed by Chou K.C. in 1999 [89]. This dataset has become a benchmark dataset for assessing AF protein features because protein members share < 30% of similarity scores, a very stringent threshold to guarantee low homology bias and redundancy in this dataset.

Marrero-Ponce et al. introduced two new types of 3D protein descriptors motivated by (1) the good performance of the most recent 2D protein bilinear indices implemented for classifying Arc mutants with high stability [148], and by (2) the success of the novel 3D molecular descriptors (QuBiLS-MIDAS) based on generalizations of geometric distances matrixes (interatomic distances) and atomic properties for modeling benchmark datasets [149,150]. The first item is the application of bilinear forms to a geometric distance matrix extended to characterize the 3D protein topology (pairwise interamino acid interactions) through the Minkowski metric [26], and the last one is the application of three linear forms as a particular case of the multilinear algebra to numerically describe the covalent and noncovalent interactions between three amino acids through three-tuple distance matrixes considering their Cα, beta-carbons (Cβ), and the average of the coordinates of all atoms in the amino acid (AVG) [151].

Both types of 3D protein descriptors reached success rates higher than 92.0% in protein secondary structural classification by using Chou’s dataset [89], being the three linear form, also called algebraic tensor’s form, the one that achieved the highest classification rate (98.18%) compared to the previously mentioned 3D bilinear descriptors, the AAC, the PseACC, the Pair coupled AA composition, and even the Position-Specific Iterative (PSI)-BLAST [151].

#### 4.2.5. ProtDCal’s Descriptors

The PROTein Descriptors CALculation (ProtDCal) program was developed to provide a diversity of protein descriptors either based solely on the sequence (0D and 1D) or on the 3D structural information. The methodology achieved such protein descriptor diversity by the combinatorial selection of different property values for the 20 natural amino acids, the application of different operators to modify the original properties values according to the vicinity of the targeted amino acid, placed either in the sequence or in the 3D structure, and the application of several clustering amino acids criteria on the modified property values. These properties were subdivided into arrays and then aggregated with different invariant operators to create a wide number of protein descriptors within 0D, 1D, and 3D families [27].

ProtDCal’s descriptors are partially inspired by the QSAR-type ones since the modification of the intrinsic index values of residues according to their particular neighborhood was performed by classic cheminformatics algorithms such as autocorrelation [152], Kier–Hall’s electro-topological state [153], Ivanshiuc–Balaban [154], and gravitational-like operators [155]. They were recently applied to discriminate enzymes and nonenzymes within the twilight zone by using the D&D benchmark dataset. The 3D structure features were ranked on the top of the Support Vector Machines (SVMs)-based methods evaluated in this dataset. On the other hand, one of the AF models using sequence-based (1D) descriptors showed a similar classification performance than other 3D structure-based methods previously evaluated in the D&D dataset. This AF (1D) model also outperformed the popular sequence-based method EzyPred [52] in detecting enzymatic signatures within a set of uncharacterized proteins of the bacterium *Shewanella oneidensis* [156].

While the ProtDCal program achieved a good performance in identifying remote homologs, it was recently developed as a suite [157] to additionally predict posttranslational modifications/sites of proteins such as N-linked glycosylation and lysine methylation sites [27,158].

#### 4.2.6. Amino Acid Sequence Autocorrelation Vectors (Descriptors)

Other QSAR-derived protein features are the amino acid sequence autocorrelation (AASA) vectors which were introduced by Caballero et al. to characterize protein sequences through the possible intramolecular interactions of their amino acids placed at different topological distance or lag (*l*) ranging from 1–15 [159]. AASA vectors are an extension of the Broto–Moreau’s formalism for estimating the autocorrelation of a topological structure (ATS) in small molecules. The ATS resulted from summing up the products of certain properties of two atoms, located at given topological distances or spatial lag in the 2D molecular graph. Then, it described the distribution of atomic properties along the topology of 2D molecular graphs. The ATS descriptors have been successfully applied to model biological activities in QSAR studies [152].

Despite AASA being applied to many bioinformatics challenges, such as the modelling of conformational stability of protein mutants in human lysozyme and gene V proteins [159,160], the prediction of dinucleotide-specific RNA-binding sites in proteins [161], and the binding stability pattern of protease-inhibitor complexes from molecular graph representation of protease sequences and ligands [162], they have not been explicitly assessed within the twilight zone for remote homology detection. Table 2 shows an updated summary of all graphical–numerical methods with their types of graphical representations and gene/protein descriptors used to detect remote homology signals. 

## 5. Ensemble of AF, AB-Based Features and Machine Learning Classification Methods for the Detection of Remote Homology in the Twilight Zone

The challenge in sequence classification [163] and specifically in remote homology detection in the twilight zone has been mainly focused in two directions: (1) The ensemble of AB and AF methods in order to merge valuable information from the primary sequence, complementing AB approaches with AF ones, and (2) the combination of various machine learning methods both to improve the precision of the classification and to cope with the knowledge extraction from big amounts of data not only in sequence datasets but also in subjacent profiles or previously curated classification by-products based on structure and function similarities.

In point (1) different approaches merge pairwise sequence comparisons with unsupervised or supervised learning classification methods representing the sequences as vectors of normalized AB score-based features plus AF-based similarity values. One example is the reference [28] with the combination of the k-NN algorithm and a weighted contribution of similarity scores, where weights reflect the discriminatory ability of individual measures in the training set. The AF similarity measures they used were the Euclidean distance and the Jensen–Shannon divergence from k-mer frequencies, and the compression-based measure built upon the concept of Kolmogorov complexity, independent of the k-mer size selection.

On the other hand, AB features were based on BLAST bit scores and Smith–Waterman-based scores in terms of P values. This integrative classification approach providing a combined sequence similarity score calculated by weighting the contribution of AB and AF sequence similarity measures improved the classification accuracy over pure AB and AF scoring schemes in predicting the taxonomic lineage for both short viral sequence fragments and complete viral sequences and in the classification of reads from a real metagenome dataset [28].

Continuing with point (1), the detection of protein remote homology has been also improved by applying the PseACC formalism to a profile-based sequence representation containing evolutionary information by estimating the amino acid occurrences for each position (column) of the MSA obtained by PSI-BLAST. Thus, the sequence order was modified according to the information of other members of the family and the resulting sequence was transformed into a vector by applying the PseAAC concept [54]. This approach of integrating AF and AB methodologies was evaluated on the well-known benchmark dataset made up by 4352 SCOP’s protein sequences with no pair with a sequence similarity higher than an *E*-value of 10^−25^ (stringent cut-off limiting homologous number) [61,164]. Liu et al. outperformed many of the state-of-the-art methodologies applied to detect remote homology when integrating AB profiles with AF protein features within the PseACC framework [54].

More recently, we addressed the remote homology detection through the identification of true orthologs within the twilight zone by combining AB and AF features under big data Spark decision tree classifiers managing imbalance between the scarce ortholog pairs and the huge amounts of nonortholog ones [29]. We reached a success rate of 98.71% on a benchmark dataset reported by Salichos and Rokas [165] consisting of yeast proteome pairs that underwent a whole genome duplication and gene losses. Previous reports on ortholog detection algorithms merging AB and AF found that just k-mers counts were considered as a first step in the ortholog and co-ortholog assignment [166]. However, in a very recent paper (2019), the author found a transitivity of similarity to construct clusters of similar proteins to avoid unnecessary comparisons in an O(*N*^2^) pairwise approach, where *N* is the total number of protein pairs. They start from the fact that combinations of sequence identity and k-mers were unsuitable for finding many homologs, just proposing a new merging cluster approach around representative sequences scalable for multigenome comparisons [167].

In a recent review of the Quest for Orthologs Consortium et al. [168], they pointed out the tendency to combine predictions from several methods in order to discover orthology relationships at large evolutionary distances, and in gene families with complex histories of gene duplications and loss, HGT, or domain gain or loss. Also, the integration of domain and gene tree information laying datasets is contributing to improve existing methods and meta-methods.

On the other hand, the strategy (2) of combining several machine learning methods or their resulting predictions was evidenced when Agüero Chapin et al. applied several sequence search methods to perform a wide-proteome exploration of the *Microcystis aeruginosa* proteome for the NRPS’s A-domain signature. They ensembled multiple-template BLASTp and profile HMM searches with the one performed by a DTM-based model built with 2D TIs from protein four-color maps encoding relevant information of A-domain sequences. Graphical profiles derived from the four-color maps and HMM profiles detected signals of the A-domain signature among the diversity of the unannotated/hypothetical proteins from the *Microcystis aeruginosa* proteome (Figure 7). Such matching predictions pointed out the existence of A-domains remote homologues in the proteome of the cyanobacteria. The integration of sequence search methods provides a higher yield for the detection of remote protein homologs with more confidence [38].

AF features and classifiers have been also integrated for improving the detection of protein remote homology [169]. Chen et al. combined the sequence composition and order within an SVM-ensemble weighted voting strategy. SVM-ensemble achieved an average 0.945 Receiver Operating Characteristic (ROC) score in a benchmark dataset by assembling SVM-based basic classifiers constructed with k-mer frequencies (SVM-kmer), auto-cross covariance (SVM-Auto-cross Covariance), and series correlation pseudo-amino acid composition (SC-PseAAC), respectively [169].

Table 3 shows examples illustrating the integration of AB and AF features into the same model or algorithm and also how AB and AF predictions from different approaches could be combined to provide a final or consensus score for detecting remote homology.

## 6. Scaling Up AB- and AF-Based Features/Measures for Homology Detection

The challenge of remote homology detection as previously mentioned can be addressed by the combination of various sequence features and/or machine learning methods to enhance sensitivity. However, this strategy implies an optimization of computing resources and data distribution mainly for feature extraction, including sequence comparison, for the preprocessing and then for the classification or clustering process. In this sense, big data solutions/platforms have been developed to address such scalability problems in bioinformatics. Since big data implementations include scalable machine learning libraries [171,172]. We mainly focus our discussion on feature extraction and/or sequence comparison stages due to their quadratic nature.

Precisely, the quadratic computational time complexity $O\left(N\;\times \;m\;\times n\right)$ of N pairwise alignment-based comparisons of sequences with m and n maximum lengths, respectively, in two comparing sets may be reduced by parallelization to $O\left(\frac{N\;\times \;m\;\times n}{p}+N\times \left(n+m\right)\right)$ if the calculation of each pair is distributed into p processors. Similarly, the order $O\left(N\;\times {\left(m+n\right)}^{2}\right)$ of the amino acid contact energy similarity calculation in aligned regions without gaps may be reduced to $O\left(\frac{N\;\times {\left(m+n\right)}^{2}}{p}+N\times \left(n+m\right)\right)$ through parallelization [173].

The scalability analysis we proposed in [173] for AB pairwise protein comparisons showed the stable ability of a parallel program to keep the efficiency in a constant value while the number of processors and the problem size were simultaneously incremented, thus achieving a kind of horizontal scalability, remarkably attainable by cloud computing with big data programs.

On the other hand, in the calculation of an alignment-free measure representing a sequence as a weighted vector X of length n, the occurrences of pattern s of length m in X may be located with $O\left(\left(n+m\right)\times logn\right)$ time complexity. In this kind of measure, vector X consists of a set of pairs $\left(c,\pi_{i}\left(c\right)\right)$ where $\pi_{i}\left(c\right)$ is the probability of character c to appear in the ith position of the sequence, with 1 ≤ i ≤ n and $\sum_{i}^{n}\pi_{i}\left(c\right)=1$. Weight $\pi_{i}\left(c\right)$ could be also modeled as the stability in the contribution of such a character c in the molecular complex [174]. Hence, the complexity of pairwise comparing calculations for such a measure may be $O\left(N\times r\times \left(n+l\right)\times \log n\right),$ where n is the maximum length of the sequences of the two comparing sets, l is the maximum length of the target patterns, and r, the total number of such patterns. If a parallel scheme is applied to pairwise comparisons that integrate various alignment-free similarity measures, then time complexity depends on the maximum complexity of all involved measures. Although the comprehensive order might be less than that of alignment-based measures, scalability would depend on the programming model and the running infrastructure.

Further data mining steps, as dimensionality reduction or feature selection, might select relevant patterns, thus reducing time complexity of subsequent sequence classification or clustering processes [172]. If, in contrast, the pairwise comparisons comprise both AB and AF features, the whole scalability might be also affected by the maximum complexity of the integrated measures and could be assured for the independent AB or AF calculation cases.

Since the beginning of big data models, the need of their application in both AB and AF sequence comparisons has arisen as stated in [175]. Indeed, solutions have arisen including CloudBLAST [176] based on MapReduce to support AB features and, similarly, alignment-free implementations, specifically one based on k-mers over Hadoop [174], to overcome not only time but also space requirements.

From the orthology or the homology inference perspectives, the computational challenge in comparing hundreds or thousands of genomes/proteomes with each other is a big data problem with solutions in progress [17]. New improvements have been developed to replace or improve BLAST by much faster homology search tools such as MMseqs2 [177] or DIAMOND [178]. Another approach is the SIBLINGS (Swiss Institute of Bioinformatics (SIB) Large INtercomparison of Genomes) project intended to provide precomputed all-vs.-all similarity scores between genes from complete genomes for further phylogenetic studies and for identifying orthologs and paralogs. This project is supported by a computationally distributed alignment supported mainly by the Swiss Institute of Bioinformatics (SIB) but other infrastructures are invited to contribute.

In total, distributed file system technologies, such as Hadoop Distributed File System (HDFS) together with machine learning libraries such as Apache Mahout running on Apache Hadoop, and MLlib on Apache Spark, can be used for big data analytics in homology detection problems to fulfill scalability in both feature extraction and sequence classification, as well as assuring efficacy of the whole data mining process [29,179].

## 7. Conclusions

The experience achieved in the last century about encoding the structure of organic compounds by applying the Chemical Graph Theory aimed to develop QSAR-type models is increasingly being transferred to analyze comparatively DNA, RNA, and proteins with no alignments. Numerous articles that report the development of new tools providing graph theory-based sequence descriptors are released each year, as well as their applications in genomics and protein science. Here, we have provided extensive evidences about their relevance for remote homology detection in real and designed datasets. At an early developing stage of the graphical–numerical methods as AF tools, they were probably not very accepted among the scientific community due to the complexity of the matrix algebra employed to numerically encode the topology of 1D, 2D, and 3D graphical maps, and because they were initially evaluated in small datasets. This is the likely explanation why they are not listed among the AF features/measures with evident applications in recent reviews.

The progressing of parallel and cloud computing empowered by the releasing of new libraries and big data platforms will undoubtedly boost the applicability of these emerging AF gene/protein descriptors at large scale. Additionally, the introduction of defined benchmark datasets recently discussed in [180] for an appropriate performance evaluation will provide more reliability to these promising sequence descriptors, which have already been integrated with “the most popular” ones and alignment-based measures to fill structural gaps for remote homology detection [29,38].

## Figures and Tables

**Table 1:** Summary of the most popular AF features applied to detect remote homology.

Word-Frequency Methods			
AF Feature	Low-Similarity Dataset	Web-Implementation	Ref.
Amino Acid Composition (ACC)	G-protein coupled receptor superfamily	COPid https://webs.iiitd.edu.in/raghava/COPid/	[46]
Pseudo Amino Acid (PseACC)	G-protein coupled receptor superfamily	http://www.csbio.sjtu.edu.cn/bioinf/PseAA/	[48,49]
PseACC	Designed dataset identity from ENZYME SwissPro database in [52]	http://chou.med.harvard.edu/bioinf/EzyPred/	[52]
PseACC	Chou’s designed dataset [89] from SCOP structural classes	http://www.csbio.sjtu.edu.cn/bioinf/PseAA/	[53]
k-mers	Benchmark Structural data designed based on [90,91]	No publicly available for proteins	[60]
k-mers	Benchmark Structural data designed in [92] and also used by [93]	No publicly available for proteins	[61]
Information theory-based methods			
Lempel-Ziv complexity	Subset of SCOP designed by [92]	No publicly available	[76]
Kolmogorov complexity	Subset of SCOP designed by [92]	No publicly available	[76]
Kolmogorov complexity (Universal Similarity Metric)	Benchmark Structural data < 25% designed based on [90,91]	No publicly available	[77]
Kolmogorov complexity (Universal Similarity Metric)	Clustering protein structures using at low sequence similarity Benchmark Structural data [91]	http://www.cs.nott.ac.uk/~nxk/USM/protocol.html	[79]

**Table 2:** Summary of the graphical–numerical features applied to detect remote homology.

Graph-Theory-Based Sequence Descriptors				
AF Feature	Low-Similarity Dataset	Graphical Representation	New Member Detected	Ref.
Stochastic spectral moments (*MARCH-INSIDE*)	RNase III family	2D Cartesian protein maps	Pac1 brk Accession DQ647826	[122]
Markovian entropies (*MARCH-INSIDE*)	Cellulase complex	2D Cartesian protein maps	-	[127]
Markovian entropies, spectral moments and electrostatic potentials (*MARCH-INSIDE*)	Mycobacterial promoters	2D Cartesian DNA maps	-	[129]
3D-Markovian descriptors (*MARCH-INSIDE*)	D&D benchmark dataset [132]	3D protein representation from PDB files considering distances between Cα of aa	-	[130]
Set of TIs for Star Networks (*S2SNet*)	Natural and unnatural proteins	2D star protein graphs		[131]
Set of TIs for Star Networks (*S2SNet*)	D&D benchmark dataset [132]	2D star protein graphs		[25]
Spectral moments (*TI2BioP*)	Bacteriocin proteins	2D Cartesian protein maps	Bacteriocin-like protein in the Cry 1Ab C-terminal domain	[134]
Spectral moments (*TI2BioP*)	RNase III family	2D Cartesian protein maps	RNase III GU190214	[135]
Spectral moments (*TI2BioP*)	ITS2 family	2D Cartesian DNA maps	ITS2 from *Petrakia sp*. FJ892749	[20]
Spectral moments (*TI2BioP*)	A-domains from NRPSs	Four-colour maps	Remote homologous in the proteome of *Microcystis aeruginosa*	[38]
3D protein bilinear indices *TOMOCOMD (QuBiLS-MIDAS)*	Chou’s designed dataset [89] from SCOP structural classes	3D PDB graphical information considering Cα and non-covalent interactions	-	[26]
3D protein three-linear indices *TOMOCOMD (QuBiLS-MIDAS)*	Chou’s designed dataset [89] from SCOP structural classes	3D PDB graphical information considering Cα, Cβ and average of the coordinates of all atoms in the amino acid	-	[151]
3D and 1D descriptors (*ProtDCal*)	D&D benchmark dataset [132]	1D Sequence information 3D PDB information		[27]

**Table 3:** Summary of the strategies combining AF and alignment-based (AB) features/measures applied to detect remote homology.

AB and AF Features/Measures Integrated under the Same Model/Algorithm			
AB/AF Features-Methods	Low-Similarity Dataset	Integrative Algorithm	Ref.
BLAST-bitscores (AB) Smith-Waterman scores (AB) k-mers (AF) Kolmogorov complexity (AF)	- Complete viral genomes - Short reads from metagenomic data [170] - Subset of SCOP designed by [92]	k-NN algorithm provides a combined score resulted from the combination/weighting of the individual scores resulting from AB and AF-based classifications	[28]
Profile-based sequence representation based on PSI-BLAST alignments Pseudo Amino Acid (PseACC)	Benchmark dataset - SCOP structural classes [61,164]	Original sequences are replaced by their profile-based representation containing evolutionary information of the family, then the PseACC concept is applied to generate AF predictors	[48,49]
Smith-Waterman (AB) Needleman–Wunsch (AB) Physicochemical profile of aligned regions (AB) ACC (AF) PseACC (AF) Composition, Transition and Distribution (AF)	Benchmark dataset reported in [165] (Saccharomycete yeast proteome pairs). Ortholog detection in the twilight zone	Decision Tree Models (DTM) implemented in the Big Data Spark platform	[29]
**Integration of Models/Algorithms Using AB and AF Features as Predictors**			
Multi-template BLASTp (AB) HMM (AB) DTM using four-colour maps (AF)	Real dataset made up of NRPS’s A-domains (10–40% of identity) and CATH domains	Assembling the predictions from AB and AF sequence similarity searches. The consensus prediction is more sensitive and reliable for detecting A-domain remote homologous.	[21,38]
Support Vector Machines (SVM) SVM-kmers (AF) SVM-Auto-cross Covariance (AF) SVM-PseACC (AF)	Subset of SCOP structural classes designed by [92]	SVM-Ensemble weighted voting strategy	[169]
